# Loss of LXRβ Drives CD4^+^ T Cell Senescence and Exacerbates the Progression of Colitis

**DOI:** 10.3390/biomedicines14010152

**Published:** 2026-01-11

**Authors:** Yang Zhang, Yalan Xu, Peng You, Yulan Liu, Jun Xu

**Affiliations:** 1 Department of Gastroenterology, Peking University People’s Hospital, Beijing 100044, China; 2 Clinical Center of Immune-Mediated Digestive Diseases, Peking University People’s Hospital, Beijing 100044, China

**Keywords:** colitis, T cell senescence, LXRβ, cGAS/STING pathway

## Abstract

**Background**: Liver X receptors (LXRs) are critical regulators of cholesterol homeostasis that modulate T cell function with anti-inflammatory effects. LXR downregulation has been implicated in the pathogenesis of inflammatory bowel disease (IBD), although its underlying mechanisms remain to be fully elucidated. Recent evidence has confirmed the link between T cell senescence and autoimmune diseases. Here, we sought to investigate whether and how LXRs regulate T cell senescence in controlling intestinal inflammation. **Methods and Results**: We found that LXRβ expression was decreased in the colons of mice with experimental colitis, and LXRβ deficiency (*Lxrβ^−/−^*) significantly aggravated their colitis. Intriguingly, this finding was accompanied by enhanced CD4^+^ T cell senescence both in the colons and spleens of *Lxrβ^−/−^* mice, evidenced by upregulation of SA-β-gal levels and the remarkable expansion of effector memory subclusters in CD4^+^ T cells. Moreover, senescent *Lxrβ^−/−^* CD4^+^ T cells secreted elevated levels of proinflammatory cytokines, especially in effector memory populations, exhibiting a pronounced proinflammatory phenotype. RNA-sequencing further confirmed the role of LXRβ in restricting CD4^+^ T cell senescence. Mechanistically, the absence of LXRβ in CD4^+^ T cells directly enhanced senescence by promoting the cGAS/STING pathway. Blocking STING signaling with a targeted inhibitor significantly alleviated senescence in *Lxrβ^−/−^* CD4^+^ T cells. **Conclusions**: Our findings demonstrate the role of LXRβ in regulating intestinal CD4^+^ T cell senescence to inhibit colitis development, identifying LXRβ as a potential therapeutic target for treating IBD.

## 1. Introduction

Liver X receptors (LXRs) are nuclear receptors that serve as vital transcriptional regulators in multiple metabolic processes, such as fatty acid, cholesterol, and phospholipid metabolism [1]. There are two LXR isoforms: LXRα and LXRβ. LXRα is predominantly expressed in the liver and adipose tissues, whereas LXRβ exhibits ubiquitous expression across various tissues [1]. Considerable evidence has demonstrated that LXRs exert anti-inflammatory effects in both innate and adaptive immune responses [2,3,4,5].

Of note, the effects of LXR-stimulated lipid metabolism on CD4^+^ T cell function have been a particular focus. Dynamic remodeling of immune-metabolic pathways, particularly lipid metabolism, represents a critical process in T cell activation [6,7]. Previous work has confirmed that LXRs can directly regulate glycosphingolipid synthesis and affect human CD4^+^ T cell function [8]. LXR activation dampened proinflammatory CD4^+^ T cell function and contributed to protective immune responses against autoimmune disorders in [9,10,11,12,13]. Furthermore, mice with T cell-specific LXRβ depletion exhibited spontaneous T cell activation due to impaired Treg cell functionality in [14]. These studies collectively demonstrated the crucial regulatory roles of LXRs in CD4^+^ T cell activation and function upon antigen recognition.

CD4^+^ T cell responses to gut microbiota are crucial for the onset of inflammatory bowel disease (IBD), which mainly encompasses ulcerative colitis (UC) and Crohn’s disease (CD) [15,16]. While the incidence of IBD is increasing globally, the etiology and underlying molecular mechanisms of IBD remain poorly understood [17,18]. Notably, a Danish case–control study showed that LXR polymorphisms implied a higher likelihood of susceptibility [19]. In addition, previous evidence has demonstrated that LXR expression is decreased in human IBD and LXRβ protects against experimental colitis in mice [20,21]. Although the anti-inflammatory effects of LXRs in intestinal epithelial cells have been clearly illustrated [20], their roles in CD4^+^ T cell function under intestinal inflammation are still largely unknown.

Recent studies have indicated that immunosenescence is related to the onset of autoimmune diseases, including rheumatoid arthritis [22] and multiple sclerosis [23]. Immunosenescence is defined as innate and adaptive immune dysfunction with aging [24]. T cell senescence may represent a key feature of immunosenescence, which is characterized by an elevated proportion of memory T cells and enhanced proinflammatory potential [25]. Although senescent T cells contribute to the progression of autoimmune disorders, the role of senescent T cells in IBD has not yet been confirmed. Interestingly, LXR-mediated cholesterol metabolism might be involved in regulating T cell senescence. When cholesterol accumulates in T cells, senescence markers and secretion of proinflammatory cytokines are elevated, while T cell cholesterol efflux can significantly suppress T cell senescence and inhibit inflammation [26]. Moreover, the preventive effects of LXRβ on cellular senescence have been confirmed [27,28].

The cyclic guanosine monophosphate (GMP)-AMP synthase (cGAS)-stimulator of interferon genes (STING) signaling pathway has emerged as a central DNA-sensing mechanism and is critically involved in the regulation of cellular senescence [29]. Intestinal mucosal damage and cellular apoptosis accompanied by ectopic DNA release may trigger the DNA-sensing cGAS/STING signaling pathway. Elevated levels of colonic cGAS and STING have been reported in human UC and CD, along with increased cell-free DNA of both nuclear and mitochondrial origin, which correlate with clinical and histological disease activity in UC [30]. Murine models of colitis also demonstrate activation of the cGAS/STING pathway and subsequent type I interferon responses. Modulation of the cGAS/STING pathway has been shown to ameliorate experimental colitis [30,31,32]. A recent study has indicated that modulation of the cGAS/STING pathway could inhibit CD4^+^ T cell senescence, thereby attenuating age-related chronic obstructive pulmonary disease [33].

Given the pleiotropic effects of activating LXRs, with their specific ligands on lipid metabolism and immune homeostasis, their preventive effects on T cell senescence are emerging as promising targets for the mechanisms underlying IBD. However, very little is known about the precise roles of LXRs in regulating immunosenescence, especially T cell senescence. The present study was thus designed to examine the effects of LXR on T cell senescence in intestinal inflammation, which might reveal insights into lipid homeostasis and immunosenescence in the progression of colitis.

## 2. Materials and Methods

### 2.1. Human Samples

Tissue specimens from the colon were obtained via endoscopy. The baseline characteristics of patients with UC and healthy donors are summarized in Supplementary Table S1. A total of 6 UC patients, diagnosed based on endoscopic, histological, and radiological criteria, were enrolled in the study. 6 healthy volunteers, free of any known acute or chronic pathological conditions, were included as controls. The study protocol was approved by the Institutional Medical Ethics Review Board of Peking University People’s Hospital (ethical approval number: 2024PHB610-001). All participants provided written informed consent prior to enrollment. Endoscopic tissue samples consisted of 1–2 mucosal biopsies collected using standard forceps from areas exhibiting endoscopic evidence of inflammatory activity in UC patients; in control subjects, normal mucosal tissues were obtained from regions adjacent to but distinct from polyps.

### 2.2. Mice

We obtained LXRβ knock-out (*Lxrβ^−/−^*) mice and wild-type (WT) mice of C57BL/6 background from Gempharmatech (Nanjing, Jiangsu, China). The primers used for genotyping of genetically modified mice are provided in Supplementary Table S2. All mice were 6–8 weeks of age at the time of experimentation and were kept in specific pathogen-free facilities with free access to a standard diet, maintained at controlled temperature and humidity, following a 12 h light–dark cycle. All the animal experiments were conducted according to guidelines that were approved by the Institutional Animal Care and Use Committee of Peking University People’s Hospital (ethical approval number: 2023PHE086).

### 2.3. Induction of Experimental Colitis

To cause the DSS-induced colitis, the mice were administered a freely available 2% dextran sulfate sodium solution (DSS, MW 40,000; MP Biomedicals, Irvine, CA, USA) for 7 days, and then normal water for 3 more days. Meanwhile, the control group was given normal water without any DSS addition. During the experiment, we monitored body weight loss, diarrhea, and bloody stools daily. The disease activity index (DAI) scores were determined based on weight loss rate, stool consistency, and bloody stool [34]. For STING inhibition in vivo, the STING inhibitor H151 was formulated in solubilizer at the concentration of 10 mg/kg and administered to the mice via intraperitoneal injection every day from the first day after 2% DSS induction.

### 2.4. Histopathological Analyses

The mice were euthanized on day 10, and colon tissues were collected for subsequent analysis. Following measurement of colon length and visual assessment, 0.5 cm segments of colon tissue were fixed in 4% paraformaldehyde and embedded in paraffin. For histopathological assessment, tissues were stained with hematoxylin and eosin (H&E). The presence of ulcers, inflammation and extent of inflammation were key parameters for assessing severity of colitis. The total colitis score was calculated as the sum of the individual scores from each parameter.

### 2.5. Colon Lamina Propria Mononuclear Cell Isolation

Lamina propria mononuclear cells from colon tissues were isolated as previously described [35]. In brief, the longitudinally opened colon tissues were washed with PBS three times and subsequently digested with RPMI 1640 medium containing 1 mM DDT, 5 mM EDTA, 20 mM HEPES, and 5% FBS (all from Thermo Fisher Scientific, Waltham, MA, USA) at 37 °C for 30 min to remove the epithelial layer. After washing in PBS three times, the remaining tissues were minced and incubated in RPMI 1640 medium containing 0.2 mg/mL collagenase IV (Sigma-Aldrich, St. Louis, MO, USA) and 0.2 mg/mL DNase I (Roche, Basel, Switzerland) for 30 min at 37 °C. The suspensions were filtered through a 70 μm strainer, and the immunocytes were separated via 30% Percoll gradient centrifugation. After washing with PBS, the purified lamina propria mononuclear cells were collected for further flow cytometric analysis.

### 2.6. Mouse CD4^+^ T Cell Isolation, Activation, and Treatment

Single-cell suspensions were obtained from mouse spleens by means of mechanical disruption and filtered through a 70 μm cell strainer. Following a 15 min incubation at 4 °C with anti-mouse CD4 magnetic beads (BD Biosciences, Franklin Lakes, NJ, USA), the cells were isolated through a magnetic cell separation system. The purified splenic CD4^+^ T cells were resuspended in complete RPMI 1640 medium. In addition to penicillin–streptomycin and 10% FBS, anti—mouse CD28 antibody (2 μg/mL) was also included. The cell suspension was added to anti-mouse CD3 antibody (5 μg/mL) pre-coated plates and cultured in a humidified incubator maintained at 37 °C with 5% CO_2_. CD4^+^ T cells were activated under specified experimental conditions, including in the presence or absence of the LXR agonist GW3965 (1 μM, 72 h; Sigma-Aldrich) [8] or the STING inhibitor H151 (1 μM, 12 h; MedChem Express, Monmouth Junction, NJ, USA) [36]. To induce cellular senescence, CD4^+^ T cells received treatment with etoposide (5 mM, 12 h; Sigma-Aldrich) [36].

### 2.7. Flow Cytometry Analysis

Isolated lamina propria mononuclear cells were suspended again in FACS buffer (PBS, 2% FBS) and blocked with an Fc block for 10 min prior to staining with antibodies specific for surface markers. All the antibodies were purchased from Biolegend (San Diego, CA, USA): anti-CD45-Alexa Fluor 700 (Clone 30-F11), anti-CD3-PE/Cyanine7 (17A2), anti-CD4-APC (GK1.5), anti-CD8-PerCP (53-6.7), anti-CD44-APC/Cyanine7 (IM7), and anti-CD62L-PE/Dazzle 594 (MEL-14). For the intracellular cytokine staining procedure, cells were first stimulated with a cocktail containing a final concentration of 50 ng/mL PMA, 1 mg/mL ionomycin, and 10 mg/mL brefeldin A for 5 h. Subsequently, cells were fixed and permeabilized with Fixation and Permeabilization Solution (BD Biosciences). After this step, we stained with the specific antibodies (all from Biolegend): anti-IL-17-FITC (TC11-18H10.1), anti-IFN-γ-PE (XMG1.2), and anti-TNF-α-Brilliant Violet 421 (MP6-XT22). We obtained all the data on the LSRFortessa (BD Biosciences) and analyzed with FlowJo 10.8.1.

### 2.8. Detecting the SA-β-Gal Activity of Immune Cells

We used flow cytometry analysis to assess senescence-associated beta-galactosidase (SA-β-gal) activity as described in a previous protocol [37]. Single cells were incubated with the fluorogenic substrate C12FDG (Invitrogen, Carlsbad, CA, USA, I-2904) for 60 min. To minimize endogenous β-galactosidase activity, pretreatment with chloroquine diphosphate was performed. Following incubation, cells were washed with PBS and co-stained with cell-type-specific markers on ice for 30 min. Cell suspensions were then immediately analyzed using the LSRFortessa flow cytometer (BD Biosciences).

### 2.9. Quantitative Real-Time PCR

We used Trizol Reagent (Invitrogen) to extract total RNA from tissues or cultured cells. The Reverse Transcriptase Kit (Thermo Fisher Scientific) was used to synthesize cDNA. In order to quantify mRNA expression, cDNAs were amplified via RT-qPCR using the SYBR Green Master Mix (Thermo Fisher Scientific). GAPDH levels were used as internal housekeeping controls to calculate mRNA relative expression. We used the Applied Biosystems StepOne Plus Real-Time PCR System to collect the data. The primer sequences for all the genes are displayed in Supplementary Table S3.

### 2.10. Western Blot Analysis

Lysates were resolved by RIPA lysis buffer supplemented with protease and phosphatase inhibitor cocktails. Then, they were shifted to PVDF membranes and probed with antibodies against P16 (#23200, 1:1000), P53 (#2527, 1:1000) (all from Cell Signaling Technology, Danvers, MA, USA), P21 (sc-6246, 1:200) (Santa Cruz, CA, USA), LXRβ (60345-1-lg, 1:1000), cGAS (29958-1-AP, 1:2000), and STING (19851-1-AP, 1:2000) (all from Proteintech, Wuhan, China). The results were normalized to those of β-actin (Servicebio, Wuhan, China), which functioned as a loading control. All data were obtained using Bio-Rad ChemiDoc (Hercules, CA, USA).

### 2.11. Immunohistochemical Staining

Colon tissues were sectioned into 4-μm-thick slices. Once deparaffinization and rehydration were carried out, the sections were blocked with 3% hydrogen peroxide. Before incubating with primary and secondary antibodies, antigen retrieval was performed. Using 3,3′-diaminobenzidine as the chromogen, immunoreactivity was visualized, and then counterstaining with hematoxylin was performed. Slides were examined under a light microscope, and positive signals were independently quantified by two investigators blinded to the experimental groups.

### 2.12. RNA-Sequencing and Analysis

To perform RNA-seq analysis, total RNA was isolated from splenic CD4^+^ effector memory T (CD4^+^ T_EM_) cells. CD4^+^ T_EM_ cells characterized by CD44^hi^ and CD62L^lo^ surface markers were sorted using a FACSAria II cell sorter (BD Biosciences). All RNA samples met quality control criteria. 1 μg of total RNA was taken as input to construct RNA-Seq libraries, which were then enriched for poly(A)-containing transcripts with the TruSeq RNA Sample Prep Kit (Illumina, San Diego, CA, USA). Next, indexed libraries were sequenced on a HiSeq 2500 platform (Illumina) following the manufacturer’s protocols, employing a 50 bp paired-end sequencing protocol. Raw sequencing data in FASTQ format were processed using in-house R scripts. The generation of gene-level read counts was performed with HTSeq version 0.11.2. We used the DESeq2 R package (1.46.0) to screen differentially expressed genes (DEGs). To enable comprehensive functional interpretation of the RNA-seq results, Gene Ontology (GO) and Kyoto Encyclopedia of Genes and Genomes (KEGG) pathway enrichment analyses were conducted to identify significantly enriched biological terms using R (version 4.6.2). Additionally, Gene Set Enrichment Analysis (GSEA) was conducted using the Java-based application provided by the Broad Institute (https://www.gsea-msigdb.org/gsea/datasets.jsp, accessed on 9 August 2024).

### 2.13. Statistical Analysis

We used GraphPad Prism 9.4.0 for all statistical analyses. Means ± standard error of measurement (SEM) was used to show quantitative data. We applied a two-tailed unpaired Student’s t-test to conduct a comparative analysis of the values between the two groups. One-way analysis of variance (ANOVA) was used to perform a statistical analysis on the data differences in more than two groups. *p*-values < 0.05 were defined as statistically significant.

## 3. Results

### 3.1. LXRβ-Deficient Mice Develop More Severe Colitis

**Figure 1:**
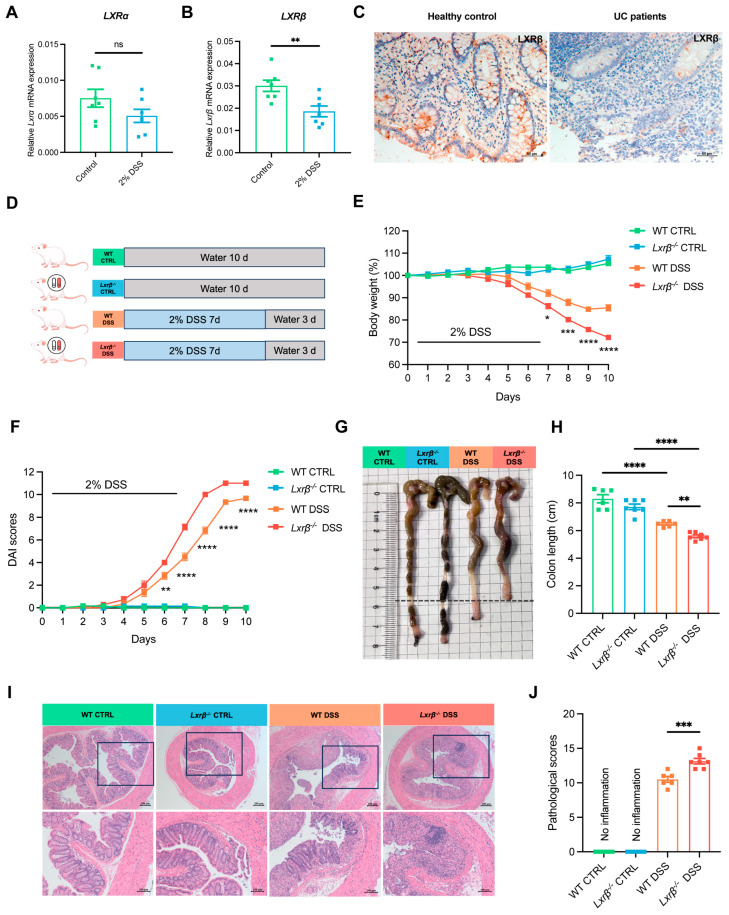
LXRβ deficiency aggravates experimental colitis. (**A**,**B**) *Lxrα* and *Lxrβ* mRNA expression in the colons of mice with DSS-induced colitis (*n* = 7 independent experiments). (**C**) Representative images of LXRβ immunohistochemical staining in endoscopic biopsies from healthy controls and inflamed mucosa from patients with UC (healthy controls, *n* = 6; UC patients, *n* = 6). (**D**) Schematic diagram of experimental colitis. The body weight (**E**), disease activity index (DAI) (**F**), and colon length (**G**,**H**) of DSS-treated WT and *Lxrβ^−/−^* mice were measured during the experimental colitis (WT group, *n* = 6 mice; *Lxrβ^−/−^* group, *n* = 7 mice). (**I**,**J**) Representative images of H&E-stained colon sections from DSS-treated WT and *Lxrβ^−/−^* mice. Scale bar: 100 μm. Images in the black box are shown at a higher magnification. Scale bar: 200 μm. The pathological scores of the colons were determined (WT group, *n* = 6 mice; *Lxrβ^−/−^* group, *n* = 7 mice). Statistical analysis of the data was conducted using a two-sided unpaired t-test (**A**,**B**,**E**,**F**) and one-way ANOVA (**H**,**J**). Data are presented as means ± SEM. * *p* < 0.05, ** *p* < 0.01, *** *p* < 0.001, **** *p* < 0.0001; ns, not significant.

A previous study showed that LXR expression in colonic tissues from patients with IBD was downregulated, and the nuclear LXR positivity was inversely correlated with histological disease activity in IBD [20]. Moreover, a DSS-induced colitis mouse model was employed to investigate the relationship between LXR and colitis. The results demonstrated that *Lxrβ* mRNA expression in the colons of mice decreased following DSS challenge, while there was no difference in *Lxrα* expression (Figure 1A,B). Another work also revealed that LXRβ had more significant anti-inflammatory effects on the colon than LXRα [21]. Additionally, immunohistochemistry staining demonstrated a lower expression of LXRβ in inflamed colon tissues from UC patients (Figure 1C). These results suggest that decreased LXR expression—especially decreased LXRβ expression—is correlated with intestinal inflammation.

To determine whether and how LXRβ regulates intestinal homeostasis, we induced colitis in WT and *Lxrβ^−/−^* mice by administering DSS (Figure 1D). First, we confirmed that *Lxrβ* expression was knocked out in *Lxrβ^−/−^* mice (Supplementary Figure S1A). We found that *Lxrβ^−/−^* mice suffered from more severe colitis and showed more weight loss (Figure 1E), higher DAI scores (Figure 1F), and more colon-shortening (Figure 1G,H), with more severe histopathological changes in the colon than WT mice (Figure 1I,J). These data indicate that LXRβ inhibits intestinal inflammation.

### 3.2. LXRβ Deficiency Contributes to CD4^+^ T Cell Senescence

**Figure 2:**
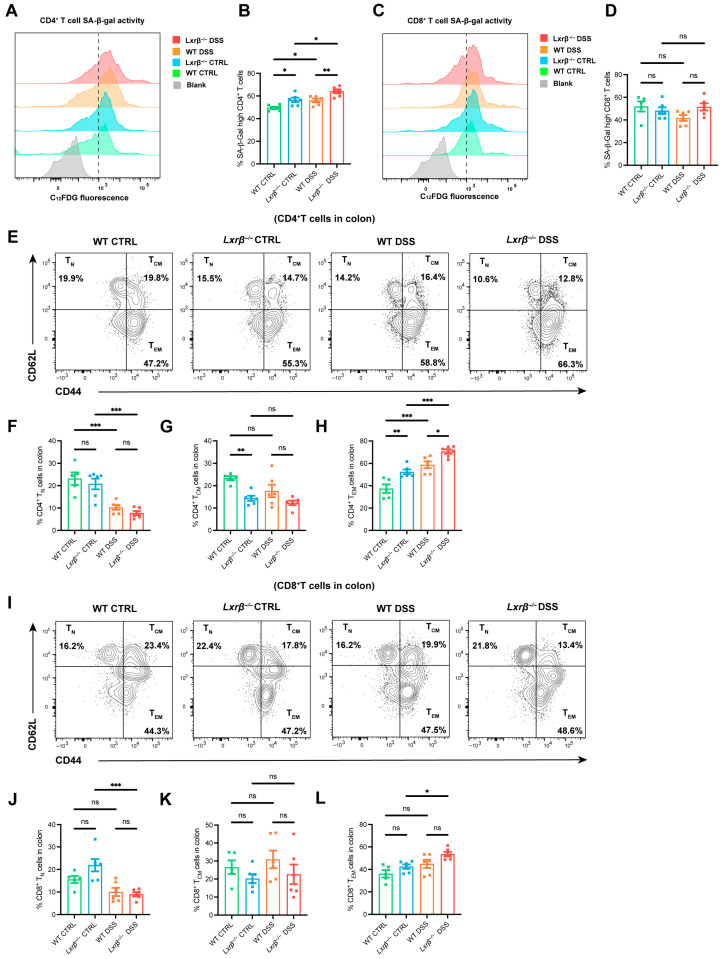
LXRβ deficiency contributes to CD4^+^ T cell senescence in the colon. SA-β-gal activity of CD4^+^ T cells (**A**,**B**) and CD8^+^ T cells (**C**,**D**) in the colon from WT and *Lxrβ^−/−^* mice, evaluated by flow cytometry using fluorescent β-gal substrate C12FDG (*n* = 6 mice per group; representative flow cytometry results are provided). Representative flow cytometry images and quantifications of CD44^−^CD62L^+^ (naïve, T_N_), CD44^+^CD62L^+^ (central memory, T_CM_), and CD44^+^CD62L^−^ (effector memory, T_EM_) subclusters in colonic CD4^+^ T cells (**E**–**H**) and CD8^+^ T cells (**I**–**L**) from WT and *Lxrβ^−/−^* mice (*n* = 6 mice per group). Statistical analysis of the data was conducted using one-way ANOVA (**B**,**D**,**F**–**H**,**J**–**L**). Data are presented as means ± SEM. * *p* < 0.05, ** *p* < 0.01, *** *p* < 0.001, ns, not significant.

To determine the role of LXRβ in T cell senescence, we analyzed cellular senescence in T cells from the colons of WT and *Lxrβ^−/−^* mice (Supplementary Figure S2A). It is widely recognized that the proportion of senescent cells increases as the proportion of naïve T (T_N_) cells decreases during immunosenescence [24]. First, we determined the proportions of senescent T cells by detecting SA-β-gal, a well-established marker of senescent cells [38]. Notably, SA-β-gal activity in various types of senescent cells is a commonly used biomarker for identifying senescence both in vitro and in vivo, and it is associated with the increased lysosome content [36,37,39]. Flow cytometry analysis of lymphocytes from the colons of these mice revealed a dramatic increase in SA-β-gal-positive CD4^+^ T cells after the DSS treatment compared with that of the control mice (Figure 2A,B); however, there was no significance in CD8^+^ T cells (Figure 2C,D), indicating that intestinal inflammation accelerated the local CD4^+^ T senescence of mucosal lamina propria. Interestingly, compared with WT mice, the *Lxrβ^−/−^* mice displayed significantly higher SA-β-gal activity in colonic CD4^+^ T cells (Figure 2A,B), but there was no significant difference in CD8^+^ T cells (Figure 2C,D). Furthermore, we did not observe any significant differences in the proportions of colon-infiltrating CD4^+^ and CD8^+^ T cells between the WT and *Lxrβ^−/−^* mice (Supplementary Figure S2B–D). The results revealed that LXRβ might provide protection against colonic CD4^+^ T cell senescence in intestinal inflammation.

As a hallmark of T cell aging, T_N_ cells decline as T_EM_ cells expand in mice and humans [25]. Next, we determined the effects of LXRβ on T subclusters. The mice administered DSS showed a significant increase in the surface expression of CD44 and reduced expression of CD62L in both CD4^+^ and CD8^+^ T cells in the colon, exhibiting an expansion of T_EM_ cells (Figure 2E–L). Additionally, colonic CD4^+^ T cells from *Lxrβ^−/−^* mice displayed more activated phenotypes, characterized by increased T_EM_ cell populations and decreased T_N_ cell populations, a phenotype resembling that observed in aged mice. However, there was no significant difference in CD8^+^ T cells (Figure 2E–L). These data demonstrated that LXRβ deficiency accelerated local activation of CD4^+^ T cells.

**Figure 3:**
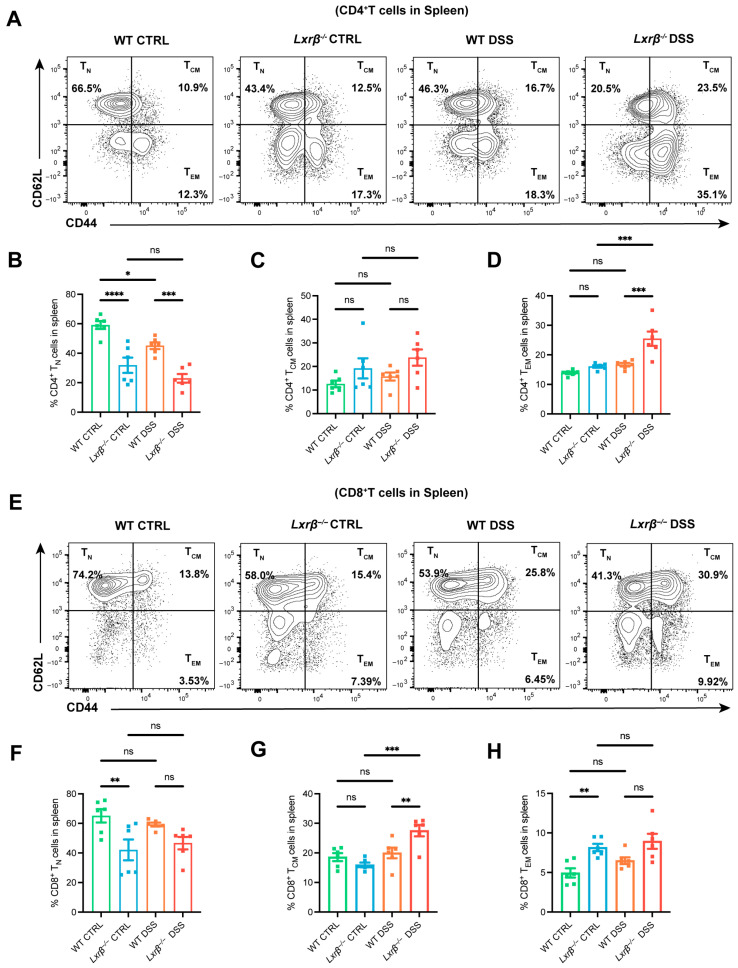
LXRβ deficiency contributes to CD4^+^ T cell senescence in the spleen. Representative flow cytometry images and quantifications of CD44^−^CD62L^+^ (T_N_), CD44^+^CD62L^+^ (T_CM_), and CD44^+^CD62L^−^ (T_EM_) subclusters in splenic CD4^+^ T cells (**A**–**D**) and CD8^+^ T cells (**E**–**H**) from WT and *Lxrβ^−/−^* mice (*n* = 6 mice per group). Statistical analysis of the data was conducted using one-way ANOVA (**B**–**D**,**F**–**H**). Data are presented as means ± SEM. * *p* < 0.05, ** *p* < 0.01, *** *p* < 0.001, **** *p* < 0.0001; ns, not significant.

Aging also results in systemic activation of T cells, which partially reflects systemic immunosenescence [25]. Next, we detected T cell subsets in the spleen (Supplementary Figure S2A). Compared with WT mice, the proportions of naive cells in the CD4^+^ and CD8^+^ T cells in the spleens of DSS-induced mice declined, and the proportions of effector memory cells increased, indicating that intestinal inflammation could accelerate the systemic immunosenescence trend. Notably, this phenomenon was amplified in *Lxrβ^−/−^* mice, which was most significant in CD4^+^ T cells (Figure 3A–H), although there was no change in splenic CD4^+^ and CD8^+^ T cell proportions (Supplementary Figure S3B–D). These observations further underscored the pervasive influence of LXRβ deficiency in shaping the senescent landscape of T cells.

### 3.3. LXRβ Depletion Promotes CD4^+^ T Cell Production of Inflammatory Cytokines

**Figure 4:**
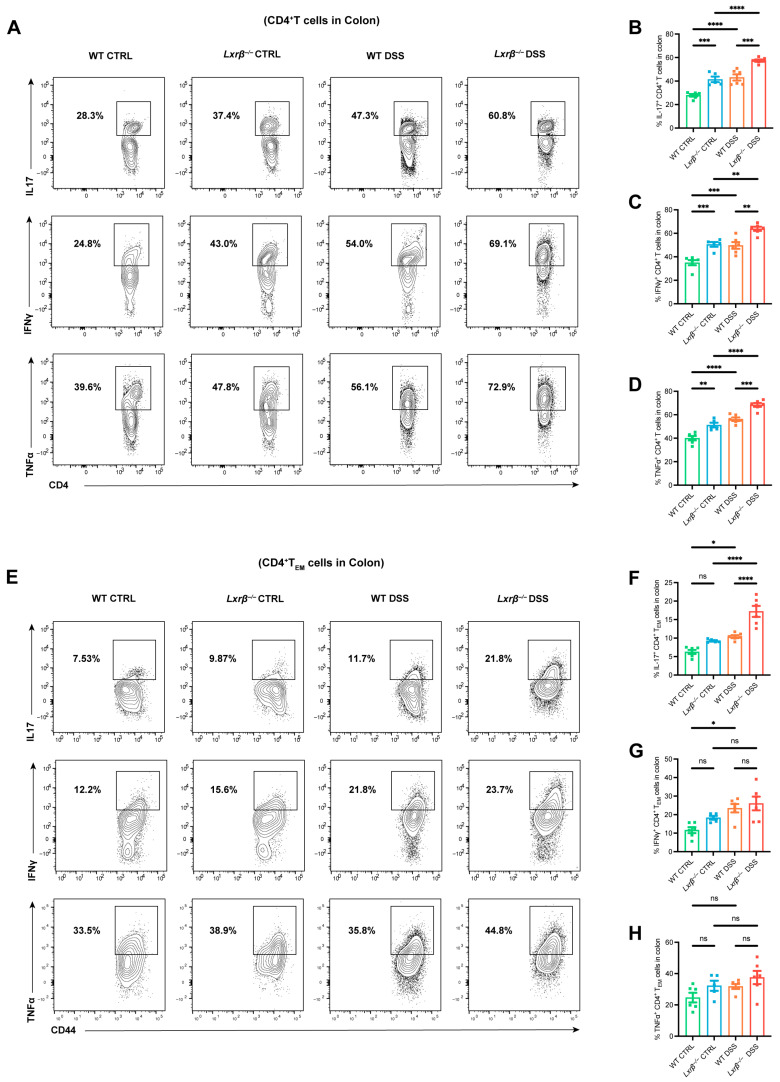
LXRβ depletion promotes CD4^+^ T cell production of inflammatory cytokines. (**A**) Flow cytometric analysis of the frequencies of CD4^+^IL-17^+^ (**B**), CD4^+^IFN-γ^+^ (**C**), and CD4^+^TNFα^+^ (**D**) cells in the colon from WT and *Lxrβ^−/−^* mice (*n* = 6 mice per group). (**E**) Flow cytometric analysis of the frequencies of CD4^+^CD44^+^CD62L^−^IL-17^+^ (**F**), CD4^+^CD44^+^CD62L^-^IFN-γ^+^ (**G**), and CD4^+^CD44^+^CD62L^-^TNFα^+^ (**H**) cells in the colon from WT and *Lxrβ^−/−^* mice (*n* = 6 mice per group). The dots within the black box denoted relevant positive cell clusters. Statistical analysis of the data was conducted using one-way ANOVA (**B**–**D**,**F**–**H**). Data are presented as means ± SEM. * *p* < 0.05, ** *p* < 0.01, *** *p* < 0.001, **** *p* < 0.0001; ns, not significant.

Senescent T cells can secrete a large quantity of proinflammatory cytokines and cytotoxic molecules [25]. Therefore, we explored the functional characteristics of CD4^+^ T cells from WT and *Lxrβ^−/−^* mice. The production of proinflammatory cytokines, including IFN-γ, TNF-α, and IL-17A, was quantified in the colonic CD4^+^ T cells. Consistent with previous reports, IFN-γ, TNF-α, and IL-17A production in total CD4^+^ T cells increased in *Lxrβ^−/−^* mice compared with WT mice (Figure 4A–D).

Previous studies have suggested that CD4^+^ T_EM_ cells might be the main source of proinflammatory cytokine production [40,41]. Therefore, we sought to determine whether CD4^+^ T_EM_ cells within the colon play a role in producing these cytokines in colitis. Intracellular staining indicated that the populations of CD44^+^IL-17^+^ subsets significantly increased, and CD44^+^IFN-γ^+^ subsets showed an increased tendency in CD4^+^ T cells from the colons of *Lxrβ^−/−^* mice compared with WT mice; however, there were no differences in the CD44^+^TNF-α^+^ subsets (Figure 4E–H). Thus, among CD4^+^ T cells in the colon, CD4^+^ T_EM_ cells might be responsible for producing these proinflammatory cytokines.

### 3.4. RNA-Sequencing Reveals Insights into Cellular Senescence in Lxrβ^−/−^ CD4^+^ T Cells

**Figure 5:**
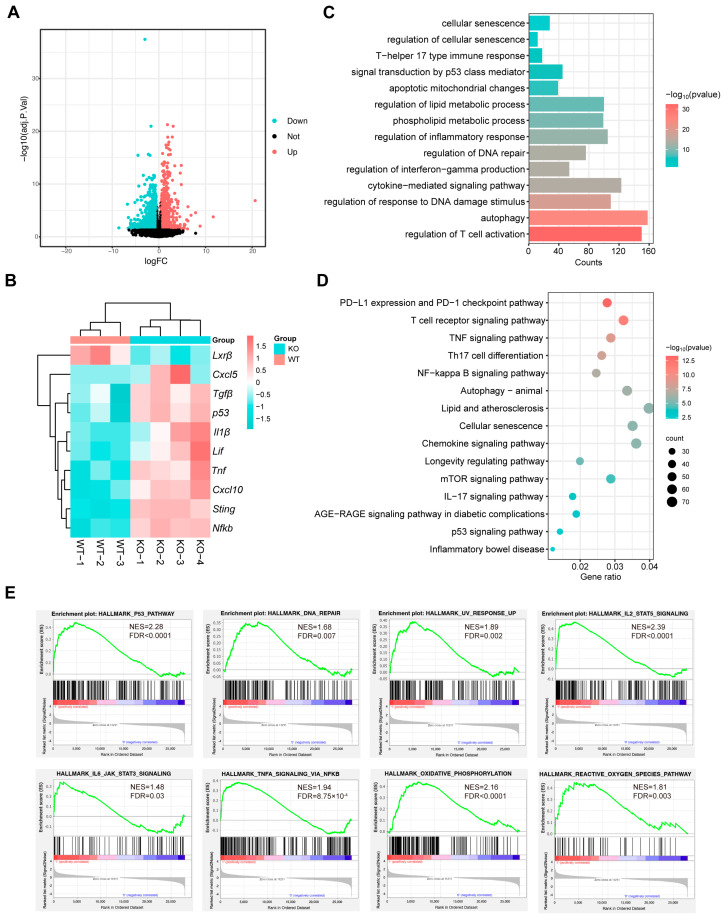
RNA-sequencing analysis of WT and *Lxrβ^−/−^* CD4^+^ T_EM_ cells. (**A**) Volcano plot illustrating DEGs between WT and *Lxrβ^−/−^* CD4^+^ T_EM_ cells. (**B**) Heatmap analysis of RNA-Seq data revealed the preferential upregulation of senescence signature genes in *Lxrβ^−/−^* CD4^+^ T_EM_ cells. (**C**) GO enrichment analysis shows the representative pathways that are enriched in *Lxrβ^−/−^* CD4^+^ T_EM_ cells. (**D**) KEGG enrichment analysis shows the representative pathways that are enriched in *Lxrβ^−/−^* CD4^+^ T_EM_ cells. (**E**) Elevated senescence-associated pathways in *Lxrβ^−/−^* CD4^+^ T_EM_ cells revealed by GSEA.

To further explore the explicit function of LXRβ in CD4^+^ T cells, we performed RNA-sequencing on splenic CD4^+^ T_EM_ cells from WT and *Lxrβ^−/−^* mice. A total of 4815 DEGs were identified (Figure 5A). Senescent cells discharge a variety of proinflammatory cytokines, chemokines, proteinases, and other bioactive factors, collectively termed the senescence-associated secretory phenotype (SASP) [42]. The SASP plays an essential role in promoting both local and systemic low-grade inflammation, contributing to age-related degenerative processes and the decline of physical function [42]. We found that LXRβ deficiency upregulated the senescence marker *p53* and accelerated the expression pattern of SASP-associated genes in CD4^+^ T cells, such as *Il1β*, *Tnf*, *Tgfβ*, *Cxcl5*, *Cxcl10*, and *Lif* (Figure 5B). Additionally, GO enrichment analysis showed that DEGs between WT and *Lxrβ^−/−^* CD4^+^ T_EM_ cells were associated with cellular senescence and senescence-associated pathways, including p53 signaling, DNA damage, autophagy, mitochondrial dysfunction, and dysregulated lipid metabolism. Moreover, the proinflammatory cytokine-associated signaling pathway and T cell immune response were also rich in *Lxrβ^−/−^* CD4^+^ T_EM_ cells (Figure 5C). Similar results were also shown by KEGG pathway enrichment analysis (Figure 5D) and GSEA (Figure 5E) of the DEGs.

### 3.5. LXRβ Deficiency Upregulates cGAS/STING Signaling to Promote CD4^+^ T Cell Senescence

**Figure 6:**
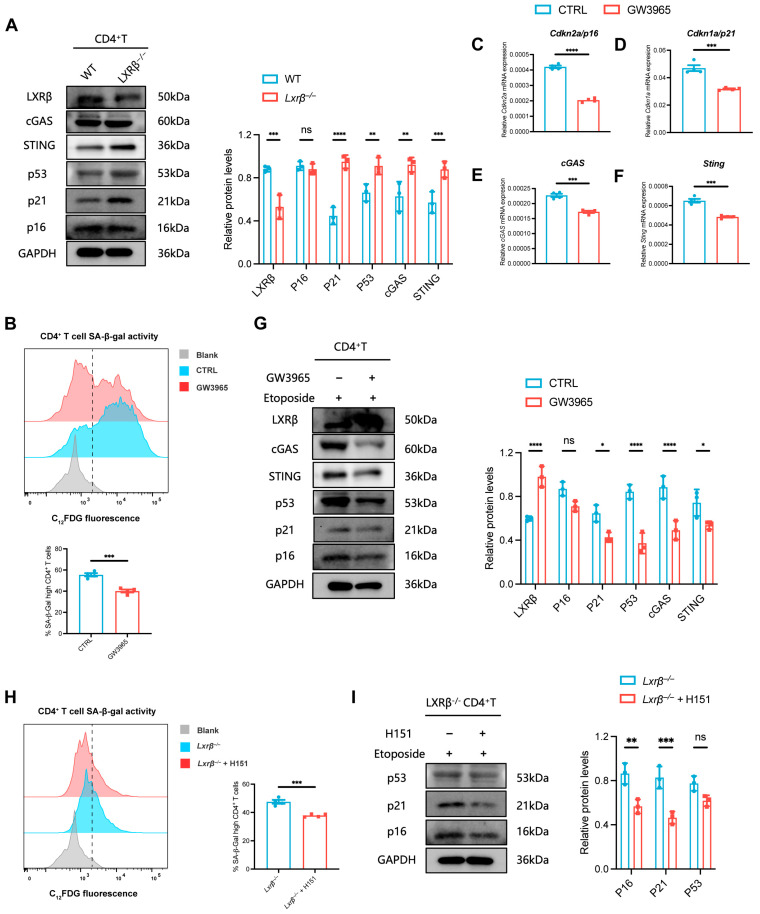
Absence of LXRβ leads to CD4^+^ T cell senescence by upregulating cGAS/STING signaling. (**A**) Immunoblot analysis of the LXRβ, senescence markers (P16, P21, P53), and cGAS/STING abundance in WT and *Lxrβ^−/−^* CD4^+^ T cells (*n* = 3 independent experiments). (**B**) SA-β-gal activity of control and GW3965-treated CD4^+^ T cells evaluated by flow cytometry using the fluorescent β-gal substrate C12FDG (*n* = 4 independent experiments; representative flow cytometry results are provided). (**C**–**G**) qRT-PCR and Western blot analysis of changes in the senescence markers and cGAS/STING mRNA and protein levels in CD4^+^ T cells after GW3965 treatment (*n* = 3 independent experiments). (**H**) SA-β-gal activity of control and H151-treated *Lxrβ^−/−^* CD4^+^ T cells evaluated by flow cytometry using the fluorescent β-gal substrate C12FDG (*n* = 4 independent experiments; representative flow cytometry results are provided). (**I**) Immunoblot analysis of the senescence markers abundance in control and H151-treated *Lxrβ^−/−^* CD4^+^ T cells (*n* = 3 independent experiments). Statistical analysis of the data was conducted using two-way ANOVA (**A**,**G**,**I**) and two-sided unpaired *t*-test (**B**,**C**–**F**,**H**). Data are presented as means ± SEM. * *p* < 0.05, ** *p* < 0.01, *** *p* < 0.001, **** *p* < 0.0001; ns, not significant.

Notably, the expression of *Sting*, a key regulator of cellular senescence, was significantly enhanced in *Lxrβ^−/−^* CD4^+^ T_EM_ cells (Figure 5B). The cGAS/STING signaling pathway has emerged as a central DNA-sensing pathway, and it is implicated in the regulation of cellular senescence [29,43,44]. A recent work identified LXR agonists as potent inhibitors of STING signaling [45]. Therefore, we sought to determine whether LXRβ regulated CD4^+^ T cell senescence through cGAS/STING signaling. First, we confirmed that loss of LXRβ contributed to CD4^+^ T cell senescence in vitro. Inhibiting LXRβ expression upregulated senescence markers (P21 and P53) and the cGAS/STING pathway in CD4^+^ T cells (Figure 6A). By contrast, GW3965, an effective agonist of LXR, could inhibit senescence marker levels and downregulate the cGAS/STING pathway in etoposide-induced senescent CD4^+^ T cells (Figure 6B–G). Furthermore, loss of LXRβ-mediated CD4^+^ T cell senescence was reversed by H151, an inhibitor of the STING pathway (Figure 6H,I). These results indicate that LXRβ deficiency promotes CD4^+^ T cell senescence via blocking restriction of the cGAS/STING pathway in CD4^+^ T cell.

**Figure 7:**
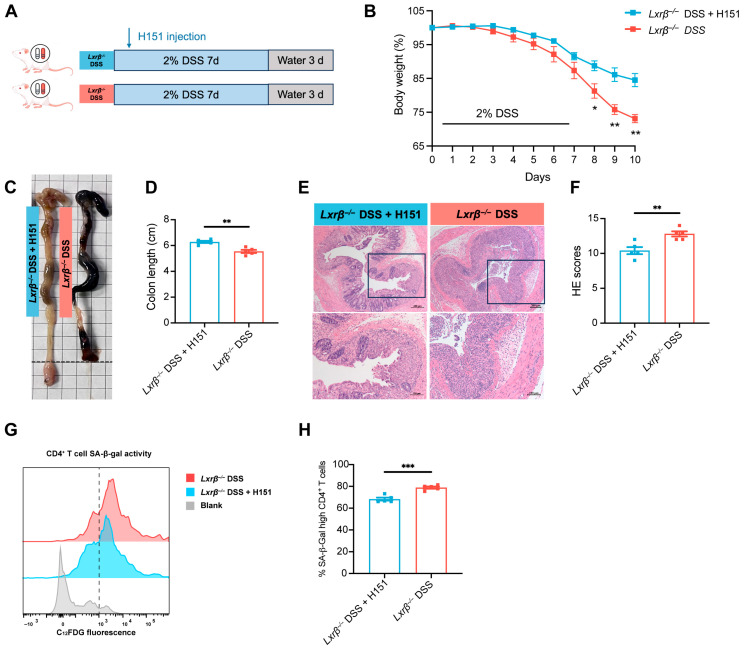
H151 ameliorates colitis in *Lxrβ^−/−^* mice by inhibiting CD4^+^ T cell senescence. (**A**) Schematic diagram of experimental colitis. The body weight (**B**) and colon length (**C**,**D**) of DSS-treated WT and *Lxrβ^−/−^* mice were measured during the experimental colitis (*Lxrβ^−/−^* + H151 group, *n* = 5 mice; *Lxrβ^−/−^* group, *n* = 5 mice). (**E**,**F**) Representative images of H&E-stained colon sections from DSS-treated *Lxrβ^−/−^* mice with or without administration of H151. Scale bar: 100 μm. Images in the black box are shown at a higher magnification. Scale bar: 200 μm. The pathological scores of the colons were determined (*Lxrβ^−/−^* + H151 group, *n* = 5 mice; *Lxrβ^−/−^* group, *n* = 5 mice). SA-β-gal activity of CD4^+^ T cells (**G**,**H**) in the colon from *Lxrβ^−/−^* mice with or without administration of H151, evaluated by flow cytometry using the fluorescent β-gal substrate C12FDG (*n* = 5 mice per group; representative flow cytometry results are provided). Statistical analysis of the data was conducted using a two-sided unpaired *t*-test (**B**,**D**,**F**,**H**). Data are presented as means ± SEM. * *p* < 0.05, ** *p* < 0.01, *** *p* < 0.001.

We further investigated whether H151 could ameliorate colitis in *Lxrβ^−/−^* mice by inhibiting CD4^+^ T cell senescence (Figure 7A). In *Lxrβ^−/−^* mice subjected to DSS treatment, administration of H151 conferred protection against severe intestinal inflammation, as evidenced by reduced body weight loss, attenuation of colon shortening, and improved histopathological outcomes (Figure 7B–F). Flow cytometric analysis revealed a decreased proportion of senescent CD4^+^ T cells infiltrating the colon (Figure 7G,H).

## 4. Discussion

**Figure 8:**
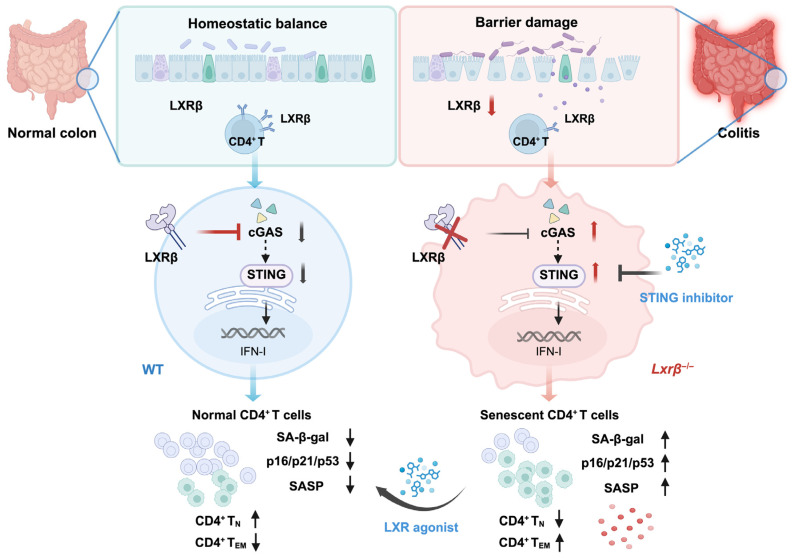
Schematic model summarizing our discoveries. We provided a link between LXRβ deficiency-mediated upregulation of the cGAS/STING signaling pathway and cellular senescence in promoting CD4^+^ T cell activation and accelerating the progression of colitis. This suggests that LXRβ upregulation may constrain CD4^+^ T cell senescence, which could be essential in maintaining intestinal homeostasis, especially under inflammatory conditions.

In the present study, we provided a link between LXRβ deficiency-mediated upregulation of the cGAS/STING signaling pathway and cellular senescence in promoting CD4^+^ T cell activation and accelerating the progression of colitis. Previous studies have shown that LXRβ activation provides protection against intestinal inflammation [20,21] and that CD4^+^ T cell function is affected by LXRβ-mediated lipid metabolism [8]. Mechanistically, we have now found that LXRβ deficiency induces CD4^+^ T cell senescence, reflected by a pronounced increase in senescence markers and a remarkable switch to effector and memory immunophenotype in CD4^+^ T cells. We also present evidence showing that LXRβ deficiency contributes to CD4^+^ T cell senescence by upregulating the cGAS/STING signaling pathway. Therefore, this study defined a novel relevant concept of LXRβ deficiency-mediated CD4^+^ T cell senescence and inflammatory response in the pathophysiology of colitis (Figure 8).

T cell senescence may represent one of the driving forces in immunosenescence. This is characterized by increased end-differentiated memory T cells and a decline in immune function accompanied by aberrant immune responses, leading to autoinflammatory and autoimmune conditions [25,46,47]. Multiple lines of evidence in human studies show that the inflammatory response mediated by senescent T cells is related to the onset of autoimmune diseases, for instance, rheumatoid arthritis [22] and multiple sclerosis [23], as well as metabolic diseases, including cardiovascular dysfunction [48] and diabetes [49]. However, the relationship between the senescence of T cells and intestinal inflammation has not yet been elucidated. Here, we revealed that senescent CD4^+^ T cells may contribute to the inflammatory response in the progression of colitis. We also identified a link between the decreased expression of LXRβ and the increased proportions of senescent CD4^+^ T cells.

To further clarify the subset composition of senescent T cells, we detected the surface expression of CD44 and CD62L in CD4^+^ T cells. We observed that effector and memory T cells increased in *Lxrβ^−/−^* mice, which was consistent with previous reports of elevated memory T cell populations during immunosenescence. Indeed, the immune system is remodeled with a decline in naïve cells and an accumulation of dysfunctional memory cells with aging, leading to greater susceptibility to infection, malignancies, and inflammatory disease [46]. The effector memory T cells become contributors to tissue damage, attacking normal tissues and inducing chronic inflammation.

Intrinsic alterations in CD4^+^ T cells have been implicated in promoting chronic inflammation and accelerating a systemic aging phenotype [50]. Senescent T cells develop a proinflammatory phenotype, and chronic inflammation is a typical phenomenon linked to immunosenescence. IBD is characterized by the chronic inflammatory environment, which may be driven by elevated production of proinflammatory cytokines, including IL-1, IL-6, and TNF-α, and acute-phase reactants released from senescent T cells. Accumulated evidence has demonstrated that effector and memory T cells are the main sources of proinflammatory cytokine production [40,41].

Cellular metabolism, including lipid metabolism, plays an essential role in controlling T cell differentiation, survival, and effector functions. Senescent T cells exhibit unbalanced lipid metabolism, which changes the expression of lipid metabolic enzymes and contributes to the accumulation of lipid droplets in T cells [51,52]. Recent evidence has indicated that T cells exhibiting elevated cholesterol levels display impaired effector function with a T cell senescence phenotype. By contrast, T cell cholesterol efflux pathways constrain T cell senescence and activation [26,52]. These studies suggest that the preventive effect of LXRβ on cellular senescence might be associated with LXRβ-mediated cholesterol efflux in T cells.

Lipid metabolism is linked to the cGAS/STING signaling pathway [53,54]. A recent work showed that stimulating lipid metabolism via LXR agonists specifically inhibited cGAS/STING signaling [45]. The cGAS-STING pathway is the primary DNA-sensing innate immune pathway, and high levels of DNA in the cytoplasm can activate the inflammatory response, diffusing and amplifying cellular immune responses. Excessive activation of the cGAS-STING signaling contributes to inflammation and senescence [29,44]. Here, the role of the cGAS-STING pathway in mediating senescence in CD4^+^ T cells was investigated. We found that inhibiting LXRβ in CD4^+^ T cells activated the cGAS/STING pathway and contributed to senescence, whereas blocking STING signaling in CD4^+^ T cells reversed LXRβ deficiency-mediated senescence. Mechanistically, LXR agonists induce the expression of sphingomyelin phosphodiesterase acid-like 3A (SMPDL3A), an enzyme capable of specifically degrading cyclic GMP-AMP (cGAMP), thereby suppressing the cGAS/STING DNA-sensing pathway. Structural analyses of crystal structures indicate that cGAMP analogs promote the dimerization of SMPDL3A, a process essential for its enzymatic activity and cGAMP degradation [45]. LXRβ agonists that target the cGAS-STING pathway have potential in attenuating CD4^+^ T cell senescence and treating inflammatory diseases. These observations extend our understanding of how lipid metabolic effectors from senescent immune cells regulate the cGAS/STING signaling pathway.

Additionally, intestinal flora dysbiosis is a core etiological factor in the pathogenesis of IBD, and LXRβ can affect flora composition by regulating intestinal epithelial barrier function. Accumulating evidence has highlighted the critical role of lipid metabolism in shaping gut microbiota [55,56]. LXR signaling pathway has been implicated in regulating gut flora and impacting metabolic-associated fatty liver disease [57]. Detecting the intestinal flora structure of LXRβ-deficient mice and analyzing the regulatory effect of flora metabolites on CD4^+^ T cell senescence is required in the future.

T cell-based immunotherapies have recently emerged as a promising and innovative strategy for the treatment of autoimmune diseases [58]. Chimeric antigen receptor T cells (CAR-T) engineered to target senescent cells have demonstrated improvements in metabolic function and physical performance in murine models under conditions of senescence overload [59]. A pivotal enzyme involved in phospholipid metabolism and the regulation of T cell senescence, group IVA phospholipase A2 (cPLA2α), has been identified in recent research as a potential therapeutic target for CAR-T cell intervention aimed at suppressing senescent T cells [51]. Furthermore, another study indicates that T cell-based therapy can alleviate systemic inflammaging and prevent senescence across multiple tissues [60]. Although the development of more specific targets for CAR-T cells directed against senescent immune cells remains ongoing, and the selective elimination of senescent cells without compromising healthy cells continues to pose a significant challenge, these findings collectively provide a strong foundation and encouragement for future investigations.

In conclusion, the present study indicated a link between CD4^+^ T cell senescence and inflammatory response in LXRβ-deficient T cells, which accelerated intestinal inflammation in the experimental colitis model. Our findings demonstrated that LXRβ deficiency induced a premature CD4^+^ T cell aging phenotype characterized by increased senescence markers and accumulated effector and memory cells. This suggests that LXRβ upregulation—for example, using an LXR agonist—may suppress CD4^+^ T cell senescence, which could be crucial in maintaining intestinal homeostasis, especially under inflammatory conditions.

However, several limitations exist in this study and warrant further research. First, the role of LXRβ expression in T cells from IBD patients and whether or not LXRβ levels correlate with T cell senescence markers in human samples remain to be explored. Incorporating human data is expected to significantly enhance the persuasiveness of the observation. Moreover, the colitis models and in vivo validation indicators for CD4^+^ T cell senescence are singular, and multi-dimensional confirmation is required. In addition, it is important to note that the potential off-target effects of pharmacological inhibitors, such as GW3965 and H151, also affect the results, warranting further validation. Finally, this study omitted the influence of gut microbiota in our experimental models. Considering the link between LXRβ, altered gut microbiota composition, and CD4^+^ T cell senescence, addressing this factor is crucial for advancing our understanding.

## Data Availability

The original contributions presented in this study are included in the article/Supplementary Material. Further inquiries can be directed to the corresponding authors.

## Supplementary Materials

The following supporting information can be downloaded at https://www.mdpi.com/article/10.3390/biomedicines14010152/s1, Figure S1: Construction of a *Lxrβ^-/-^* mouse experimental model. (A) Each mouse was validated by two pairs of primers (first band size, target 405 bp KO 2749 bp; second band size, target 402 bp KO 0bp). The position of the first and second mouse gene samples is about (400 bp, none), which is homozygous (i.e. *Lxrβ^-/-^* mouse), the position of the sixth and seventh mouse gene samples is about (none, 400 bp), which is WT mouse, and the position of other mouse gene samples are about (400 bp, 400 bp), which is heterozygous (i.e., *Lxrβ^+/-^* mouse); Figure S2: Gating strategy for flow cytometry in the colon. (A) Representative flow cytometric plots of gating strategies for colon lamina propria lymphocytes staining panels. (B) Representative flow cytometric plots and percentages of colonic CD4^+^ T cells (C) and CD8^+^ T cells (D) from WT and *Lxrβ^-/-^* mice (n = 6 mice per group). Statistical analysis of the data was performed using one- way ANOVA (C,D). Data are presented as means ± SEM. * *p* < 0.05, ** *p* < 0.01, *** *p* < 0.001, **** *p* < 0.0001; ns, not significant; Figure S3: Gating strategy for flow cytometry in the spleen. (A) Representative flow cytometric plots of gating strategies for splenic lymphocytes staining panels. (B) Representative flow cytometric plots and percentages of splenic CD4^+^ T cells (C) and CD8^+^ T cells (D) from WT and *Lxrβ^-/-^* mice (n = 6 mice per group). Statistical analysis of the data was performed using one- way ANOVA (C-D). Data are presented as means ± SEM. * *p* < 0.05, ** *p* < 0.01, *** *p* < 0.001, **** *p* < 0.0001; ns, not significant; Table S1: Clinical characteristics of 6 UC and 6 healthy controls. Table S2: The primers for identification of LXRβ knock-out mice; Table S3: The primers for RT-qPCR.
