# Supplementary material for: Loss of LXRβ Drives CD4+ T Cell Senescence and Exacerbates the Progression of Colitis
Source: Biomedicines. 2026 Jan 11;14(1):152. doi: 10.3390/biomedicines14010152 (PMC12838642; doi:10.3390/biomedicines14010152)
Supplement: Supplementary file 1 [file biomedicines-14-00152-s001.zip › Supplementary Table S1.pdf]

Clinical characteristics of 6 UC patients and 6 healthy controls.

**ID**	**Gender**	**Age(years)**	**Disease extent**	**Site of biopsies**	**Indication for colonoscopy**	**Histology**
HC1	Male	42	-	rectum	physical examination	normal colonic mucosa
HC2	Female	33	-	rectum	physical examination	normal colonic mucosa
HC3	Male	38	-	rectum	physical examination	normal colonic mucosa
HC4	Male	44	-	rectum	physical examination	normal colonic mucosa
HC5	Female	36	-	rectum	physical examination	normal colonic mucosa
HC6	Male	40	-	rectum	physical examination	normal colonic mucosa
UC1	Male	35	E1	rectum	flare of UC	active chronic colitis
UC2	Female	31	E2	rectum	flare of UC	active chronic colitis
UC3	Female	41	E1	rectum	flare of UC	active chronic colitis
UC4	Male	43	E1	rectum	flare of UC	active chronic colitis
UC5	Male	41	E1	rectum	flare of UC	active chronic colitis
UC6	Female	32	E3	rectum	flare of UC	active chronic colitis
