# Supplementary material for: Loss of LXRβ Drives CD4+ T Cell Senescence and Exacerbates the Progression of Colitis
Source: Biomedicines. 2026 Jan 11;14(1):152. doi: 10.3390/biomedicines14010152 (PMC12838642; doi:10.3390/biomedicines14010152)
Supplement: Supplementary file 1 [file biomedicines-14-00152-s001.zip › Supplementary Table S2.pdf]

The primers for identification of LXRβ knock-out mice.

Primer	Forward primer (5'-3')	Reverse primer (5'-3')	Band size
**T017369-1**	AACAAGGTGGGCGTGACTCTAC	CAACATGATCTGGAGGTGACAAGG	WT: 2749bp KO: 405bp
**T017369-2**	CAGGGTCCCATCCAACAAGGTA	CAACATGATCTGGAGGTGACAAGG	WT: 402bp KO: 0bp
