# Supplementary material for: Loss of LXRβ Drives CD4+ T Cell Senescence and Exacerbates the Progression of Colitis
Source: Biomedicines. 2026 Jan 11;14(1):152. doi: 10.3390/biomedicines14010152 (PMC12838642; doi:10.3390/biomedicines14010152)
Supplement: Supplementary file 1 [file biomedicines-14-00152-s001.zip › Supplementary Table S3.pdf]

The primers for RT-qPCR.

Genes	Forward primer (5'-3')	Forward primer (5'-3')
Cdkn1a/p21	CCTGGTGATGTCCGACCTG	CCATGAGCGCATCGCAATC
Cdkn2a/p16	CGCAGGTTCTTGGTCACTGT	TGTTCACGAAAGCCAGAGCG
cgas	AATGATACAGCGCAGCGAGA	CTGCAACAACCCATGCAACA
Sting	GCCCTGTCACTTTTGGTCCT	TGGAGTATGGCATCAGCAGC
Lxrα	AGGAGTGTCGACTTCGCAAA	TGTAGACCATGTAGTTGAGGTCA
Lxrβ	CATTGCGACTCCAGGACAAGA	CCCAGATCTCGGACAGCAAG
