# Supplementary figures and images for: Loss of LXRβ Drives CD4+ T Cell Senescence and Exacerbates the Progression of Colitis
Source: Biomedicines. 2026 Jan 11;14(1):152. doi: 10.3390/biomedicines14010152 (PMC12838642; doi:10.3390/biomedicines14010152)

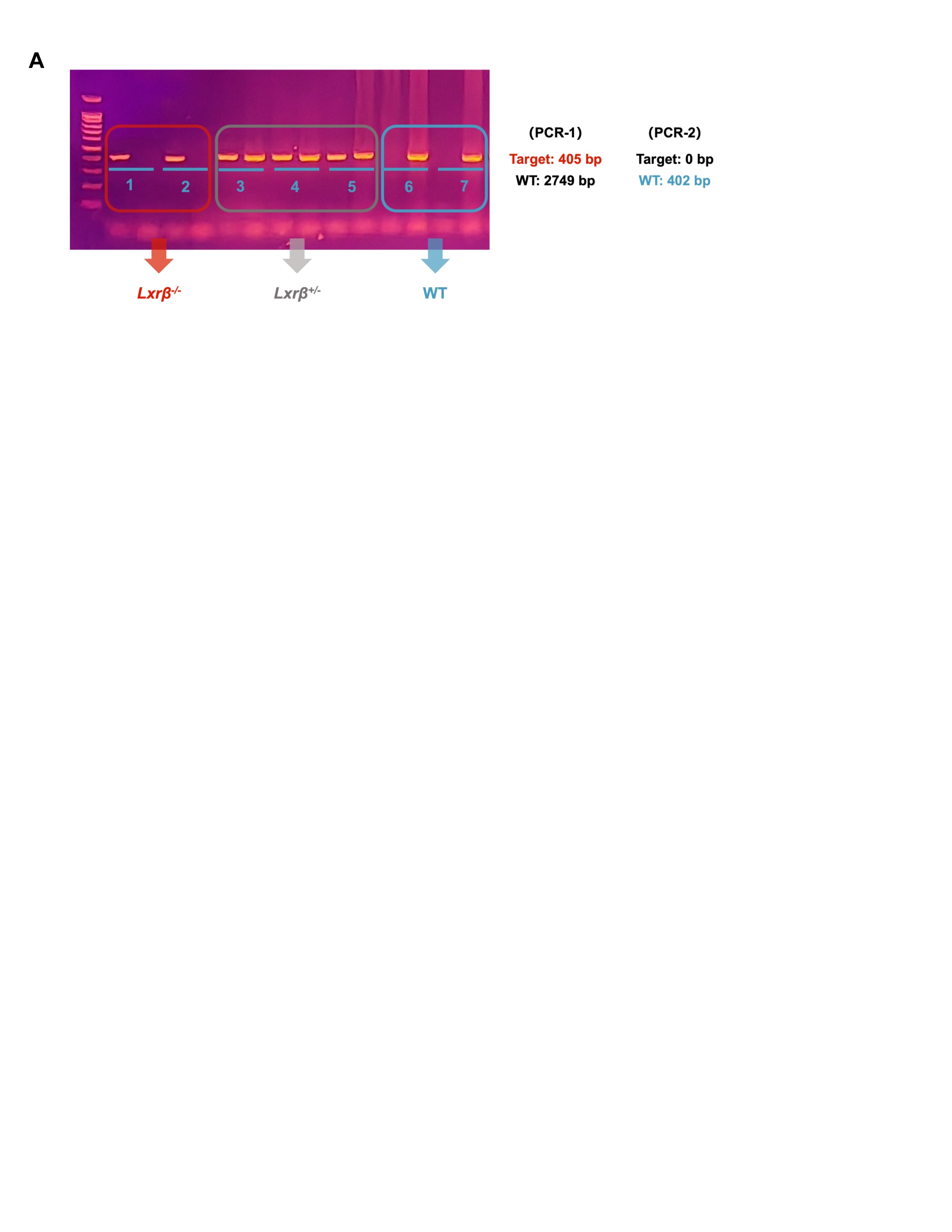

Supplement: Supplementary file 1 [file biomedicines-14-00152-s001.zip › Supplementary Figure S1.tif]

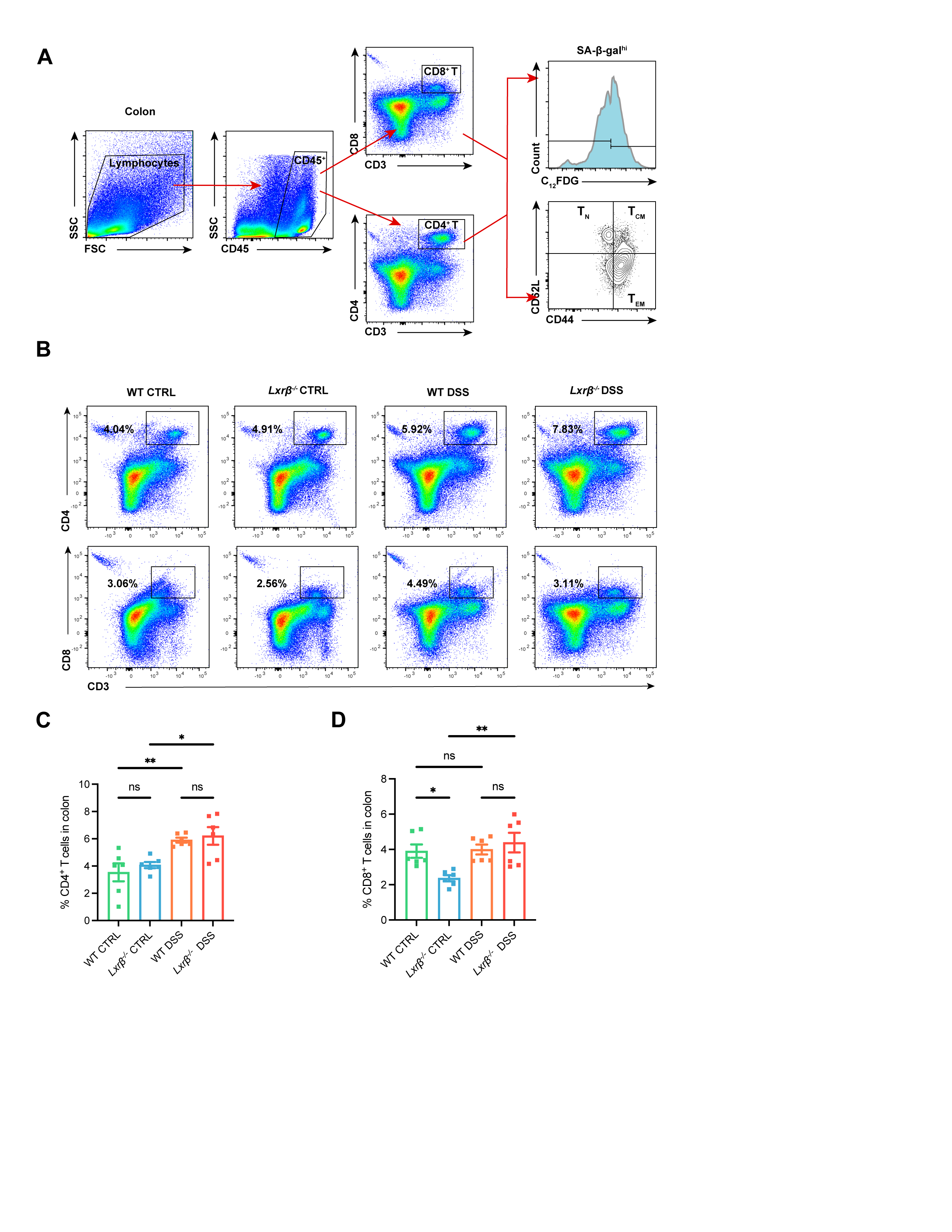

Supplement: Supplementary file 1 [file biomedicines-14-00152-s001.zip › Supplementary Figure S2.tif]

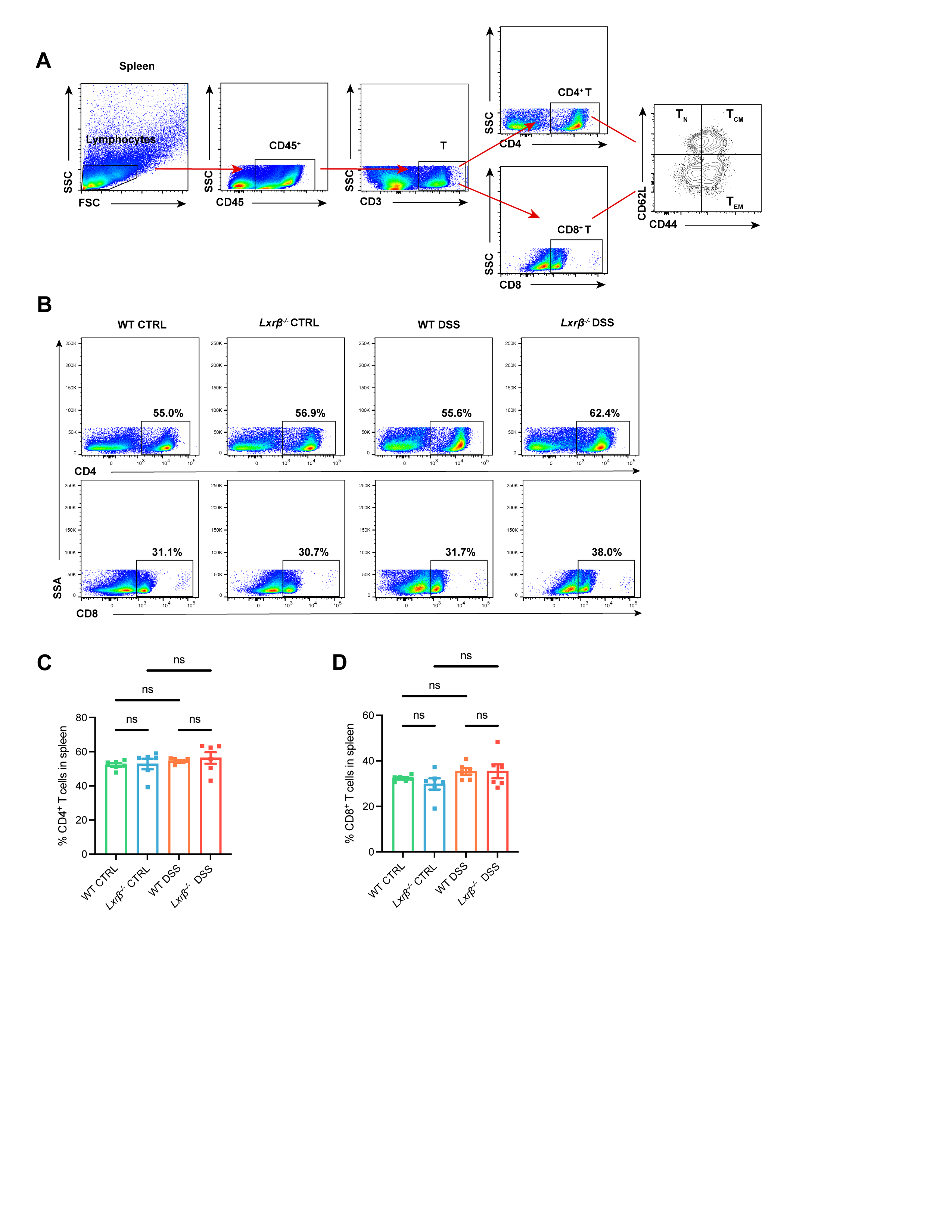

Supplement: Supplementary file 1 [file biomedicines-14-00152-s001.zip › Supplementary Figure S3.tif]
